# Screening of the Nutritional Properties, Bioactive Components, and Antioxidant Properties in Legumes

**DOI:** 10.3390/foods13223528

**Published:** 2024-11-05

**Authors:** Mihaela Multescu, Alina Culetu, Iulia Elena Susman

**Affiliations:** National Institute of Research & Development for Food Bioresources, IBA Bucharest, 6 Dinu Vintila Street, 021102 Bucharest, Romania

**Keywords:** legumes, hemp, amino acids, protein digestibility, total polyphenolic content, flavonoids, antioxidant capacity

## Abstract

This study provides an assessment of nutrients (protein, amino acid profiles, fiber, starch), phenolic content TPC, flavonoid content TFC, and antioxidant capacity through different in vitro methods in 12 legume species (red, green, yellow, brown, and black lentils; mung, pinto, black, and kidney beans; chickpea, soy, and lupin) and hemp. Legumes with a protein content above 30% were black lentil, lupin, and soy. Chickpea, soy, black bean, kidney bean, and mung bean did not have any limiting amino acids. All samples had moderate overall protein quality, except green and brown lentils. Black bean was less digestible (68.1%), while soy, hemp, and red lentil had higher protein digestibility (79.3–84.7%). Pinto bean had the highest TPC (425.19 mg GAE/100 g), comparable with hemp, but the lowest TFC (0.24 mg QE/100 g). Yellow and red lentils showed the lowest TPC (69–85.89 mg GAE/100 g). Mung bean presented the highest concentration of flavonoids (45.47 mg QE/100 g), followed by black lentil (28.57 mg QE/100 g). There were distinct variations in the antioxidant capacity across different legume samples and assays. Pinto bean, hemp, and green lentil had the highest relative antioxidant capacity index, while yellow lentil, red lentil, and chickpea presented the lowest. Dark-colored legume samples showed a higher TPC and a lower antioxidant capacity (CUPRAC and PCL assays), while yellow legumes had less antioxidant capacity (DPPH assay). A high correlation coefficient was observed between TPC and DPPH (r = 0.8133), TPC and FRAP (r = 0.8528), TPC and CUPRAC (r = 0.9425), and TPC and ACL (r = 0.8261) methods. The results highlight large variations in the legume properties and support the exploitation of the nutritional properties of legumes as raw materials for the development of products designed to fulfil modern consumer demands.

## 1. Introduction

As a response to global food concerns due to the rapid growth of the global population, which is estimated to reach 10.4 billion people by 2100 [1], research is being invested in expanding the food industry’s resources. One direction is the widespread use of legumes in the food sector.

The legume family (*Fabaceae* or *Leguminosae*) is one of the largest plant families. Legumes have the potential to contribute to healthy and sustainable human diets due to their high content in protein (17–45%), fiber (12–48%), and other bioactive compounds [2,3,4,5]. Legume proteins are deficient in sulfur-containing amino acids, but they contain a high amount of the essential amino acid lysine, thus being complementary to cereal proteins (which contain adequate amounts of methionine and cysteine and are limited in lysine). Legumes represent a good environmentally friendly alternative to animal-based products for human consumption, providing a viable source of protein for people who want to reduce meat consumption [2,6]. Legumes also contain anti-nutritional factors, such as trypsin inhibitors, phytates, lectins, saponins, and phenolic compounds, which, at certain doses, affect human health [7,8]. Therefore, several methods (among them, germination, fermentation, cooking, soaking, or toasting) are used to reduce the concentration of anti-nutritional factors [9,10].

Legumes as raw materials play a significant role in the food industry due to their nutritional and physicochemical properties. Baked foods, snacks, legume-based beverages, and gluten-free products have been developed for general consumers as well as for those with specific dietary considerations (celiac patients, vegetarians, vegans, diabetics) [2]. Thus, nowadays, research is directed at the incorporation of legumes into diverse types of innovative products with the aim of promoting legume consumption and enhancing the human diet [5,11,12]. Alongside the nutritional aspects, legumes have a great impact on ecosystem services in terms of lower greenhouse gas emissions and increased soil fertility and crop production through the fixation of atmospheric nitrogen [3,13].

An important subcategory of legumes is represented by different varieties of beans, soybeans, peas, lentils, chickpeas, lupins, carobs, and peanuts [11].

Soy is one of the best-known legumes in the world, and it has a high nutritional value (around 40% protein, essential amino acids, and other beneficial nutrients) [3,14]. Another nutritionally healthy legume is lentils. They are a rich source of protein (25.8%), carbohydrates (60%), dietary fiber (30%), and bioactive compounds, such as flavonoids, polyphenols, tannins, saponins, and protease inhibitors [15,16]. An increase in bean intake is related to health-promoting effects and protection against some diseases (cardiovascular, diabetes, cancer). This is mainly due to the great number of polyphenols and other compounds with antioxidant activities [17]. Also, the consumption of chickpeas can protect against several diseases due to their nutritional quality [18]. Chickpeas have high amounts of protein (17–30%), fiber (18–22%), polyunsaturated fatty acids (about 66%), and minerals and vitamins [18,19]. Lupin is an important grain legume that has received attention recently due to its characteristics. Alongside the protein content (30–50%), lupin seeds are a rich source of non-starch polysaccharides (30–40%), unsaturated fats (5–15%), and minerals and have low anti-nutrient contents (e.g., phytic acid, lectins, and saponins) [20]. Fechner et al. [21] showed that some lupine components in food reduce total and LDL cholesterol in the blood.

The aim of this paper was to evaluate and investigate the nutritional profile in terms of proximate composition, amino acid profile, phenolic and flavonoid content, and antioxidant capacity for different types of legumes available on the market for future development of new food products. The legume samples in this study were lentils (red, green, yellow, brown, black), beans (mung, pinto, black, kidney), chickpea, soy, and lupin. Even though hemp is not a legume, we included it in this study for comparison due to its valuable protein and potential for the food industry.

## 2. Materials and Methods

### 2.1. Legumes Sources

Thirteen legume samples available on the market were evaluated. Green, black, brown, and yellow lentils, as well as red kidney, black, and pinto beans, were from Bioplanet (Leszno, Poland). Mung bean, hemp, and chickpea were from BiOrganik (Budapest, Hungary). Red lentil, white lupin, and soy were acquired from Kotys (Brasov, Romania), Primeal (Peaugres, France), and Bauckhof (Rosche, Germany), respectively. For the analysis, the legume samples were milled (MultiDrive control, IKA, Staufen, Germany), and the obtained powders were stored in airtight brown glass jars at room temperature.

### 2.2. Chemical Composition Analysis

Composition analysis was performed following the AOAC International method [22]. Briefly, moisture content was analyzed gravimetrically via sample drying in an oven at 105 °C (AOAC 925.10), while ash content was analyzed via sample burning in a muffle furnace at 550 °C (AOAC 923.03). Protein content was determined by the Kjeldahl method with mineralization and distillation unit (Foss Analytical, Höganäs, Sweden) (AOAC 920.152), and fat was extracted with a Soxtec system (model 2055, Foss Analytical Höganäs, Sweden) using petroleum ether (AOAC 922.06). Total dietary fiber was measured via the enzymatic–gravimetric method (AOAC 991.43) using K-TDFR kit (Megazyme International Ltd., Bray, Ireland). Starch was quantified via the AOAC 996.11 method with K-TSTA kit (Megazyme International Ltd., Bray, Ireland).

### 2.3. Amino Acid Composition Analysis

Approximately 0.1 g sample was hydrolyzed in 5 mL 6 M HCl at 110 °C for 12 h. After hydrolysis, the samples were filtered and evaporated, and the dried amino acids were reconstituted in 0.5 mL water. Then, the samples were filtered through a 0.45 µm syringe filter and analyzed using a 1260 Infinity II high-performance liquid chromatography system (Agilent Technologies, Waldbronn, Germany). For amino acid analysis, a Poroshell HPH-C18 column (4.6 × 100 mm, 2.7 µm; Agilent) and guard column (4.6 × 5 mm, 2.7 µm) were used with an automatic pre-column derivatization method using o-pthalaldehyde and 9-fluorenylmethyl chloroformate, according to the standard protocol of the manufacturer [23]. An AdvanceBio amino acid reagents kit (Agilent) was used for calibration of the amino acids in the range 10–1000 pmol/µmol. Tryptophan was not determined as it is decomposed during acid hydrolysis.

For the evaluation of the nutritional quality of protein, the amino acid score (AAS), protein digestibility-corrected amino acid score (PDCAAS), and essential amino acid index (EAAI) were calculated. AAS represents the ratio of the amount of a certain essential amino acid in the sample to the amount of the same amino acid in the reference pattern for older children, adolescents, and adults as established by FAO [24]. PDCAAS was calculated by the product between the lowest AAS value and the percentage of the protein digestibility of the sample [25]. EAAI was calculated as the geometric mean of the ratio of all essential amino acids in the samples to those in the standard egg protein [26].

### 2.4. Determination of In Vitro Protein Digestibility

The in vitro protein digestibility of the legume samples was determined according to Hsu et al. [27]. An amount of 50 mL of aqueous protein suspension comprising 6.25 mg protein/mL was prepared at pH 8.0 (adjusted with 0.1 M NaOH). Trypsin (1.6 mg/mL; type IX-S, Sigma-Aldrich, St. Louis, MO, USA) was used as a hydrolyzing enzyme, and all the reaction conditions took place at 37 °C in a water bath under constant stirring. The pH change after 10 min was used to calculate the percentage of in vitro protein digestibility (Y) with the equation Y = 210.46 − 18.10 · X, where X represents the sample pH after 10 min in vitro digestion.

### 2.5. Color Analysis

The color of legume samples was analyzed using Minolta CM-5 colorimeter (Konica Minolta Sensing, Inc., Osaka, Japan). CIE L*a*b* color coordinates were determined using the standard light source D65 with the 10° observer angle and a shutter of 30 mm. Measurements were conducted in 10 different points of the sample, and average color parameters were reported for the following: L* (lightness coordinate), a* (red-green component), and b* (yellow-blue component of a color). Based on the values of L*, a*, and b* color coordinates, the chroma (C*) color attribute was calculated.

### 2.6. Preparation of Methanolic Extracts for Bioactive Compounds and Antioxidant Capacity

For the preparation of methanolic extracts, approximately 0.1 g of the sample was placed in a 50 mL centrifuge tube, and then 10 mL of 80% methanol were added. The extraction was performed on a vortex (Multi Reax, Heidolph Instruments, Schwabach, Germany) at maximum speed for 4 h at room temperature. Subsequently, the extracts were centrifuged at 10,000 rpm for 30 min, and the supernatant was recovered for further analysis (Section 2.7, Section 2.8, Section 2.9, Section 2.10, Section 2.11, Section 2.12 and Section 2.13).

### 2.7. Determination of Total Polyphenolic Content (TPC)

TPC measurement was carried out using the conventional Folin–Ciocalteu method. Briefly, 500 μL of 80% methanolic extract reacted with 5 mL of the Folin–Ciocalteu phenol reagent (diluted with water 1:15). The reaction was developed for 10 sec, and subsequently, 500 μL of 20% sodium carbonate were added [28]. The blue color complex was allowed to develop for 20 min; after this time, the absorbance readings were carried out at 752 nm in a spectrophotometer (Specord 210 UV-VIS, Analytik, Jena, Germany). A standard curve was prepared using different concentrations (0.005–0.175 mg/mL) of gallic acid under the same condition as the samples (R^2^ = 0.9999). The results were expressed in terms of mg of gallic acid equivalent per 100 g of sample dry basis (mg GAE/100 g d.b.).

### 2.8. Determination of Total Flavonoid Content (TFC)

The TFC was measured following a procedure described earlier [28]. Briefly, 500 µL of sample extract in 80% methanolic solution was mixed with 1500 µL 95% ethanol, 100 µL of 10% aluminum chloride hexahydrate, 100 µL of 1 mol/L sodium acetate, and 2.8 mL water. Absorbance readings were recorded at 415 nm after 30 min incubation under dark conditions at room temperature. A standard curve was plotted using different concentrations (0.02–0.30 mg/mL) of quercetin (R^2^ = 0.9994). The flavonoid content in the samples was expressed in equivalent quercetin mg per 100 g sample dry basis (mg QE/100 g d.b.).

### 2.9. Determination of Antioxidant Capacity Through DPPH Method

The antioxidant capacity of the radical 2,2-diphenyl-1-picrylhydrazyl (DPPH) was carried out according to a previous method [28]. An amount of 400 µL of extract reacted with 6 mL of the DPPH radical (0.04 mg/mL). Absorbance readings at 517 nm were carried out after 30 min of reaction under dark conditions. Antioxidant capacity was calculated using a calibration curve (0.05–0.60 mM) obtained with Trolox (6-hydroxy-2,5,7,8-tetramethylchroman-2-carboxylic acid) (R^2^ = 0.9995). Antioxidant capacity values were expressed as mg Trolox equivalent per 100 g sample dry basis (mg TE/100 g d.b.).

### 2.10. Determination of Antioxidant Capacity Through ABTS Method

ABTS (2,20-azino-bis(3-ethylbenzothiazoline-6-sulphonic acid) diammonium salt) assay was performed according to Horszwald and Andlauer [28]. The reaction mixture consisted of 200 μL of sample and 3 mL of ABTS^•+^ working solution. After 6 min of incubation at room temperature, the absorbances were measured at 730 nm. The standard curve was linear between 0.025 and 0.25 mM Trolox (R^2^ = 0.9999). The results were expressed in mg Trolox equivalent/100 g sample dry basis (mg TE/100 g d.b.).

### 2.11. Determination of Antioxidant Capacity Through FRAP Method

The ferric ion-reducing antioxidant power (FRAP) assay was measured as stated earlier [28]. A 2700 µL aliquot of a solution containing a mixture of 5 mL 2,4,6-tri(2-pirydyl)-s-triazine TPTZ (10 mM in 40 mM HCl), 5 mL ferric chloride (20 mM in water), and 50 mL sodium acetate (300 mM adjusted to pH 3.6) was added to 450 µL of sample extract. Absorbance readings were carried out at 593 nm. Antioxidant capacity was calculated using a calibration curve between 0.25 and 100 µM Trolox (R^2^ = 0.9998). The results were expressed in mg Trolox equivalent per 100 g sample dry basis (mg TE/100 g d.b.).

### 2.12. Determination of Antioxidant Capacity Through CUPRAC Method

Copper ion reduction was performed according to the method described by Celik et al. [29]. Thus, 100 μL of sample/standard solutions of different concentrations was mixed with 1 mL CuCl_2_ (10 mM), 1 mL neocuproine (7.5 mM), and 1 mL ammonium acetate buffer 1 M, pH = 7.00. After 30 min, the absorbance was measured at 450 nm. The required Trolox stock solutions for the calibration curve were 10 mM, and the working concentrations were between 50 and 2000 μM (R^2^ = 0.9996). The results were expressed in mg Trolox equivalent per 100 g of sample dry basis (mg TE/100 g d.b.).

### 2.13. Photochemiluminescence (PCL) Assay

The assay described by Popov and Lewin [30] was commercially distributed as a comprehensive system known as Photochem^®^ by Analytik Jena AG (Jena, Germany). The PCL-ACL (lipid-soluble antioxidant capacity) protocol was used. For the reactions, kits designed for determining the antioxidant capacity of lipid-soluble substances were utilized. The reaction mixture consisted of 2300 μL of methanol (reagent 1), 200 μL of buffer solution (reagent 2), 25 μL of luminol (reagent 3), and 50 μL of sample extract. The measurement was carried out using the Photochem device with PCLSoft 5.1 software. A calibration curve was prepared using Trolox. The results were expressed as mg of Trolox equivalent per 100 g of sample dry basis (mg TE/100 g d.b.).

### 2.14. Development of the Relative Antioxidant Capacity Index (RACI)

The relative antioxidant capacity index (RACI) was used as a statistical perspective by integrating the antioxidant capacity data determined through the various methods used. In order to obtain the RACI values, the standard scores for each method were determined according to a previous described method [31].

### 2.15. Statistical Analysis

The analyses were performed in triplicate and the results were expressed as means ± standard deviation. Statistical analysis was performed using one-way analysis of variance (ANOVA), followed by Tukey’s test with significant differences at *p* < 0.05 (Minitab software, version 19, Minitab Inc., Coventry, UK). The Pearson correlation was used to determine the relation between results.

## 3. Results and Discussion

### 3.1. Chemical Composition of Legume Samples

The chemical composition showed significant (*p* < 0.05) variations among legume samples (Table 1) for protein, fat, ash, starch, and fiber content. The protein concentration was significantly higher (*p* < 0.05) in hemp (47.25%), soy (43.86%), and lupin (42.24%) compared with lentils (28.19–33.23%), beans (22.36%–28.34%), and chickpea (24.80%). Among lentils, black lentil had the highest protein content (33.23%). Yellow, red, brown, and green lentils (protein in the range of 28.2–28.9%) and kidney bean (28.34%) had significantly higher protein content (*p* < 0.05) than black (24.79%), pinto (23.42%), and mung (22.36%) beans. Similarly, black lentils showed higher protein content compared with other lentil species (28.4% vs. 24.4–25%) [32], while Sinković et al. [33] reported lupins with the highest protein content (32–43%) among pulses. Crude protein contents of 20–24% for common beans, 26–28% for lentils, and 19% for chickpea were also declared [33].

The fat level was significantly higher (*p* < 0.05) in soy (24.36%), hemp (10.42%), and lupin (7.45%) compared with the other samples. Brown lentil had the lowest fat content (0.66%). Also, green lentil, black lentil, and mung bean had fat content < 1%. On the other hand, red lentil and yellow lentil, as well as pinto, black, and kidney beans, had higher fat content in amount of 1.2–1.4%, while chickpea showed higher fat content (5.7%). Sinković et al. [33] found a fat content between 5–9% in lupins and chickpeas, which aligns with the present results.

The ash values were in the range of 2.5–4.2% for most of the analyzed samples, except soy and hemp, with higher value of 5.2% and 8.5%, respectively. Overall, bean samples had higher ash content than lentils, lupin, and chickpea.

The fiber content showed variability between samples and decreased in the following order: green lentil > lupin > pinto bean > yellow lentil > kidney bean > hemp > chickpea > black lentil > mung bean > black bean > brown lentil > red lentil > soy. Previous research in analyzing lupin, pea, chickpea, grass pea, lentil, and bean showed that the fiber content in legume seeds differ significantly in different species and their varieties [34].

Starch is a major nutrient in the investigated legumes, with a lower amount in lupin, soy, and hemp (<20%).

Differences in the physicochemical properties of legumes are due to a combination of factors related to varieties, environmental conditions (weather effects, light intensity, temperature, or geographical location), and agronomic practices (soil fertility or weeds), as well as, to a certain extent, the methods of analysis used [5,35].

When legumes are subjected to different types of treatments, the nutrients content undergoes changes compared with the raw legumes. For example, cooking and soaking lowers the protein, fiber, fat, and ash content in cooked lentil and chickpea [16,18]. On the other side, the germination process shows an increase in the protein content in germinated chickpeas, cowpeas, lentils, and yellow peas than the non-germinated examples [36]. In addition, an increase in the content of protein, fiber, and carbohydrate was found in germinated red lentils and black beans [37]. Also, fermentation produces positive effects on the nutritional quality of legumes, with increased protein content and reduced fat content [38]. However, the increasing effect on the nutritional parameters is dependent on the conditions employed during germination (duration, temperature, substrate) and fermentation (temperature, time, pH, fermentation type, microorganism strain, substrate), which contribute to the activation of endogenous enzymes.

### 3.2. Amino Acid Composition

The quality of a protein depends on its amino acid profile and digestibility [39]. Table 2 compares the amino acid profiles of the legume samples. The two most abundant amino acids were glutamic acid (15.25–27.61%) and aspartic acid (10.43–13.91%), while the lowest levels were associated with sulfur-containing amino acids methionine (0.82–1.6%), and cysteine (0.65–1.49%). The relatively low levels of sulfur-containing amino acids are a characteristic of legumes [34,40]. Among legume samples, chickpea had the highest content (*p* < 0.05) of methionine (1.6%) and cysteine (1.49%). The percentage of cysteine was similar for chickpea, soy, and hemp (*p* > 0.05). The methionine content in hemp was significantly higher (*p* < 0.05) than in the legume samples. Overall, chickpea, soy, and hemp met the FAO requirements for sulfur-containing amino acids (>2.5%).

For all analyzed samples, the essential amino acids leucine, valine, isoleucine, threonine, histidine, and aromatic amino acids (phenylalanine + tyrosine) met the requirements recommended by FAO [24]. In addition, all legumes met the lysine requirements (>4.8%), whereas lupin just met the lysine requirements, and hemp (with a lysine content of 4%) did not meet the FAO’s requirements.

Red lentil was characterized by higher contents of leucine (8.43%), lysine (7.68%), phenylalanine (5.79%), valine (5.06%), isoleucine (4.81%), threonine (4.54%), histidine (2.93%), aspartic acid (13.91%), alanine (4.77%), and serine (5.89%). In contrast to red lentil, green lentil had the lowest content of the above-mentioned amino acids. Soy and lupin had similar leucine contents to red lentil (*p* > 0.05). Chickpea, mung bean, soy, and pinto bean had similar phenylalanine contents to red lentil (*p* > 0.05), while the lowest content was for lupin, brown lentil, and green lentil (4.62%). The variability among the legume samples for valine content ranged from 3.97% to 5.06%, isoleucine content from 3.92% to 4.81%, and threonine content from 3.39% to 4.54%. Overall, the lowest essential amino acids content was for green and brown lentils. Gorissen et al. [40] stated that the lower content of essential amino acids may contribute to the lower anabolic capacity of the protein.

Changes in amino acid concentration occur during different types of legume treatments. The heating process and fermentation contribute to the degradation of large protein molecules to small peptides and amino acids [41]. Fermentation’s impact on the amino acid profile differs, mainly because of variations in the initial protein content and the amino acid profile of each legume, as do the specific fermentation conditions (pH, type of microorganism, strain, and substrate used) [38]. On the one hand, the content of basic amino acids (lysine and arginine) was reduced because of low pH values used during bean fermentation; but on the other hand, the content of threonine, leucine, isoleucine, phenylalanine, valine, and methionine was increased [42]. Methionine increase was also reported in germinated legumes [37]. Moreover, microwave cooking, autoclaving, and microionization increased the sulfur amino acid content in several types of legumes (cowpeas, beans, peas) [43]. Considering the deficient sulfur amino acids content found in unprocessed legumes, different treatments applied to legumes bring different benefits to them. However, optimum processing conditions are necessary for each type of legumes to maximize the nutrition advantages provided by several treatments used.

The results of the amino acid scores are presented in Table 3. When a value of the AAS is lower than 1, the corresponding amino acid is considered a limiting one. Thus, lentil samples, pinto bean, and lupin showed a lack of cysteine and methionine compared to the FAO reference protein, while hemp had a limited content of lysine. In addition, chickpea, soy, black bean, kidney bean, and mung bean did not have any limiting amino acids (as AAS > 1).

PDCAAS indicates the ability of protein to provide the required essential amino acid for the human body. The closer the value is to 1, the better the quality of protein in the sample. In this study, PDCAAS ranged between 0.50 and 0.92 (Table 3), with the lowest values for all the lentil samples. Soy, chickpea, and mung bean had the highest PDCAAS values and consequently had a higher nutritional value of amino acids compared with the other investigated sources. Regarding the bean samples, pinto bean had a lower quality than mung, kidney, and black beans. Lupin exhibited an appropriate PDCAAS value with hemp, and higher than that of the lentil samples.

EAAI assesses the overall protein quality of the sample, and it does not refer to certain amino acids, like PDCAAS. The higher the EAAI, the more balanced the amino acid composition and the higher the quality of the protein. Based on the EAAI values, the protein sources are classified as high nutritional value (EAAI is between 86 and 95), moderate protein source (EAAI is between 75 and 86), and unsuitable protein source (EAAI is less than 75) [26]. Table 3 shows that green and brown lentils had a low protein quality (the lowest EAAI), while all the other samples had a moderate protein quality.

In general, the values of the protein quality indexes are improved as different types of legume treatments have increasing effects on the amino acid composition [43].

### 3.3. Protein Digestibility

**Figure 1:**
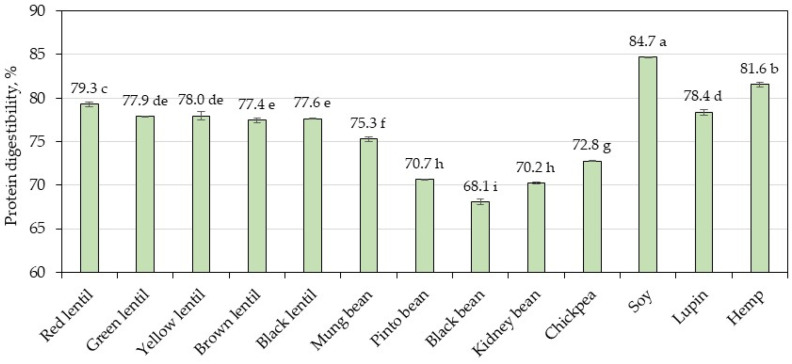
In vitro protein digestibility values. Bars with different letters represent a significant difference between samples (*p* < 0.05).

The in vitro protein digestibility of the legumes samples is reported in Figure 1. Black bean had significantly lower digestibility (68.1%) than all the other legumes samples studied; soy had the highest (84.7%); and the others were in a range of 79.3–70.2%. Hemp had a protein digestibility of 81.6%, significantly higher (*p* < 0.05) than all the legumes, with the exception of soy. Within lentil samples, there were no significant differences in in vitro protein digestibility (*p* > 0.05) between green, yellow, brown, and black lentils, while red lentil showed significantly higher values (*p* < 0.05). Lupin was in the range of the lentil samples. Bean samples and chickpea sample had the lowest digestibility (*p* < 0.05).

The in vitro protein digestibility of the analyzed legumes was compared with the total amino acid content, resulting in a correlation of r = 0.5729 (*p* < 0.05), indicating that the samples with higher total amino acid content showed higher digestibility. Protein digestibility refers to the extent to which a protein is broken down into its constituent amino acids, making them available for absorption within the gastrointestinal tract.

Proteins from legumes and cereals present low digestibility (75–80%) compared to the protein coming from animal sources, which have high digestibility (>95%) [41]. The lower digestibility of plant protein is attributed to several factors, such as protein structure (mainly β-sheet conformation), permeability of cell walls, formation of complexes, and anti-nutritional factors [25,38,44]. Among the anti-nutritional factors, protease inhibitors, tannins, and phytates have the greatest impact on digestibility. Thus, protease inhibitors inhibit the activity of trypsin and chymotrypsin [44], while tannins bind proteins by forming tannin–protein complexes [38] and thus reduce protein digestibility in legumes. In addition, phytate–protein complexes inhibit protein digestibility by reducing protein solubility [44]. However, processing legumes leads to an increase in protein digestibility [32]. Methods such as thermal treatments, toasting, extrusion, fermentation, germination, ultrasonication, high-pressure processing, microwave treatment, irradiation, or enzymatic hydrolysis are used as strategies to improve the in vitro digestibility of plant proteins [8,45].

### 3.4. Bioactive Compounds Content

Phenolic and flavonoid compounds are essential for health, offering antioxidant, anti-inflammatory, and antimicrobial benefits [46]. The TPC and TFC of the analyzed legumes are presented in Table 4. The TPC ranged from 69 to 425.19 mg GAE/100 g d.b. On an overall assessment, hemp powder exhibited the highest TPC value of 432.56 mg GAE/100 g d.b., comparable to pinto bean (*p* > 0.05), whereas yellow and red lentils showed the lowest content (69–85.89 mg GAE/100 g d.b.). In terms of lentil powders, green lentils presented the highest concentration of polyphenolic compounds, followed by black and brown lentils. There was no significant difference in TPC (*p* > 0.05) between black and brown lentils, with a content around 248 mg GAE/100 g d.b. Yellow lentils showed the lowest concentration of polyphenolics. With respect to bean extracts, pinto bean extract revealed the greatest concentration of phenolics (425.19 mg GAE/100 g d.b.), while black bean showed the smallest TPC content (209.53 mg GAE/100 g d.b.). All bean samples were significantly different (*p* < 0.05) in terms of TPC. No significant difference was found in TPC between brown lentil, black lentil, and soy, as well as between red lentil and chickpea (*p* > 0.05).

Several studies have compared the phenolic content in different legumes. Green lentil exhibited the highest TPC (0.96 mg GAE/g f.w.), followed by black lentil (0.84 mg GAE/g f.w.), brown lentil (0.81 mg GAE/g f.w.), and red lentil (0.79 mg GAE/g f.w.) [47]. Additionally, Durazzo et al. [48] reported that green lentil exhibited the highest TPC values (737.32 mg GAE/100 g d.w.) compared to red lentils (373.89 GAE/100 g d.w.). Other lentils varieties, namely, Pardina and Crimson, had TPC of 11.8–12.0 mg GAE/g, much higher than that of chickpea (2.2 mg GAE/g) and soybean (2.3 mg GAE/g) [49]. In different mung bean cultivars, TPC ranged from 2.05 to 2.38 mg GAE/g [50]. Black bean showed a TPC of 176.3 mg GAE/g d.m., and pinto bean showed a TPC of 222.5 mg GAE/g d.m. [51]. Another study found lower contents of total phenols (0.47–2.38 mg GAE/g d.w.) in 26 kidney beans [52]. Giusti et al. [53] reported a content of 3.00 mg GAE/g d.w. for kidney bean and 2.24 mg GAE/g d.w. for cranberry bean, although much lower contents were found for kidney (0.96 mg GAE/g d.w.) and black (0.90 mg GAE/g d.w.) beans [54]. In regular-darkening cranberry beans, the total phenolic content was higher (2.82 to 4.15 mg GAE/g) than in non-darkening varieties (0.67 to 0.81 mg GAE/g) [55]. Mastura et al. [56] found that TPC in raw organic beans ranged from 268.99 mg GAE/100 g in mung beans to 453.77 mg GAE/100 g in black beans. For raw inorganic beans, Adzuki beans had the highest TPC at 395.38 mg GAE/100 g, while black beans had the lowest value at 238.04 mg GAE/100 g. In other legumes, TPC varied based on factors like cultivation methods and years of harvest. For example, Carbas et al. [57] found fluctuations in TPC in beans between 0.11 and 4.59 mg GAE/g depending on the harvest year. Mekky et al. [58] found that TPC in seven Egyptian chickpea cultivars ranged from 69 to 129 mg GAE/100 g. In three lupin species, TPC varied from 212.12 to 317.88 mg GAE/100 g [59]. Hemp samples had a variability in TPC, ranging from 1368 to 5160 mg GAE/100 g d.m. [60], while other studies reported lower levels, i.e., 3.56 and 3.76 mg GAE/g d.m. for two industrial hemp cultivars [61], and 135.90 and 198.88 mg GAE/100 g for two varieties from Morocco [62].

The total flavonoid content (TFC) in the analyzed legumes varied from 0.16 mg QE/100 g d.b. to 45.47 mg QE/100 g d.b. (Table 4). Mung bean presented the highest concentration of flavonoids, followed by black lentil (28.57 mg QE/100 g d.b.) and lupin (26.22 mg QE/100 g d.b.). Chickpea and pinto bean had the lowest TFC (*p* > 0.05). With respect to lentil samples, the content of flavonoids decreased (*p* < 0.05) in the following order: black lentil > green lentil > brown lentil > red lentil > yellow lentil. For beans samples, mung bean exhibited the highest TFC (45.47 mg QE/100 g d.b.), while pinto bean showed the lowest flavonoid content (0.24 mg QE/100 g d.b.). Kidney bean and hemp did not present significance difference in TFC, with a value around 0.2 mg QE/100 g d.b. (*p* > 0.05).

The flavonoid content in 21 bean samples from Portugal ranged from 0.80 to 4.33 mg CE/g d.m. (2013) and 0.91 to 4.02 mg CE/g d.m. (2014) [57]. Red lentils had the highest TFC (0.06 mg QE/g) among lentils, while green lentils had the lowest (0.02 mg QE/g) [47]. Kabuli chickpeas from Turkey showed a TFC between 114.3 and 118.1 mg CE/100 g [63]. The TFC in soybean varieties from Croatia ranged from 0.428 to 0.659 mg CE/g d.m depending on year of harvest [64]. TFC in hemp from Morocco varied between 39.40 to 69.54 mg QE/100 g [62]. Also, Rashid et al. [65] reported flavonoid contents from 34.52 to 47.12 mg QE/100 g in hemp, which was lower than in earlier reports.

All the above-presented studies show the variation in TPC and TFC in the same type of samples. This may be attributed to varietal differences, climatic conditions, harvest timing, and other factors influencing the nutritional quality of the plants. Additionally, the solvent used in the extraction for analysis plays a significant role in determining the TPC and TFC levels [66].

The quantity and type of phenolics and flavonoids are important, but their relative importance depends on the specific health outcome being targeted [67]. In terms of processing, cooking can reduce the content of sensitive compounds, while simultaneously favoring the formation of simpler phenolics from complex ones [68]. Germination generally boosts both the quantity and effectiveness of polyphenol compounds, as through the activation of various enzymes, the protein–bound polyphenol complexes are gradually released, enhancing their availability and potential health advantages [36]. Fermentation also contributes to an overall increase in TPC [38,42]. In general, processing conditions are key factors in controlling the nutritional improvement of legumes.

### 3.5. Antioxidant Capacity by Different Assays

The response of antioxidants can vary depending on the specific type of radical or oxidant source. Therefore, no single method can fully capture the mechanisms of action for all radical sources or all antioxidant compounds within a complex system [69]. For estimating the total antioxidant capacity, using different assays is crucial for determining the overall antioxidant potential of any food matrix. The measurements provided by DPPH, ABTS, FRAP, and CUPRAC assays are presented in Table 5.

The values of antioxidant capacity using the DPPH method ranged between 63.48 and 600.00 mg Trolox/100 g d.b., with the highest value for pinto bean, followed by green lentil (480.46 mg Trolox/100 g d.b.) and kidney bean (395.63 mg Trolox/100 g d.b.). For lentils, yellow lentil exhibited the lowest antioxidant capacity (*p* < 0.05), while green lentil presented the greatest value of DPPH. For the bean samples, the DPPH values were in a range of 158.73 mg Trolox/100 g d.b. for mung bean to 600 mg Trolox/100 g d.b for pinto bean. It is observed that yellow lentil exhibited the lowest total phenolic content, which corresponds to the lowest DPPH scavenging activity.

No significant differences were found in DPPH values between lupin and red lentil and hemp and black lentil (*p* > 0.05). On the other hand, the DPPH activity was significantly different in both lentil and bean samples (*p* < 0.05).

The radical scavenging activity, measured via the ABTS assay for the analyzed legumes, ranged from 245.97 to 2499.54 mg Trolox/100 g d.b. (Table 5). The ABTS values for soy and lupin were the highest, while yellow lentil had the lowest (*p* < 0.05) ABTS activity, which was associated with the lowest total phenolic content. Among the lentil samples, green lentil demonstrated the highest ABTS activity, followed by black, red, and yellow lentils. For the bean samples, pinto bean displayed the highest antioxidant capacity (1149.57 mg Trolox/100 g d.b.), while black bean recorded the lowest value (587.82 mg Trolox/100 g d.b.). ABTS values in all samples were significantly different (*p* < 0.05), except mung bean and green lentil (*p* > 0.05).

The antioxidant capacity of legumes, determined via the FRAP method, ranged from 43.64 to 351.62 mg Trolox/100 g d.b. (Table 5). The highest FRAP value was observed in green lentil (*p* < 0.05), whereas yellow lentil, red lentil, and chickpea samples had the lowest antioxidant capacity (*p* > 0.05). Durazzo et al. [48] also found higher FRAP values for green lentil compared with red lentil (140.32 μmol/g d.m. vs. 66.37 μmol/g d.m.). As for the bean samples, pinto bean demonstrated the highest FRAP value at 332.20 mg Trolox/100 g d.b., followed by kidney bean and black bean.

Regarding the CUPRAC antioxidant assay, hemp presented the highest capacity (1208.31 mg Trolox/100 g d.b.), and among the legumes, pinto bean was the highest (1093.65 mg Trolox/100 g d.b.) (Table 5). The highest CUPRAC activity in hemp corresponds to the highest TPC. A wide range of CUPRAC values was observed among the lentil extracts, showing values of 298.43–1060.27 mg Trolox/100 g d.b., with the lowest value for red and yellow lentils. For bean samples, the CUPRAC values were between 524.62 mg Trolox/100 g d.b. and 1093.65 mg Trolox/100 g d.b.

There is great variability in the antioxidant activities of legumes between studies. In a study evaluating 11 lupin samples, the antioxidant capacity using DPPH, ABTS, and FRAP assays was in the range 0.99–1.88 μmol TE/g d.m., 5.50–7.92 μmol TE/g d.m., and 1.33–6.07 μmol TE/g d.m., respectively [70]. In chickpea genotypes from different countries, the antioxidant activity had a wide interval from 39 to 1650 μmol TE/100 g d.w. (DPPH), from 278 to 2418 μmol TE/g d.m. (ABTS), and from 41 to 1083 μmol TE/g d.m. (FRAP) [71]. Another study highlighted the difference in the antioxidant activities through DPPH, ABTS, and FRAP assays in several bean cultivars from two harvest years with different climatic conditions [57].

As for the TFC and TPC results, the variations in antioxidant capacity results observed among different studies are attributed to factors related to seed cultivation, including varietal characteristics, climate change, agrotechnical practices, and storage conditions, as well as the methods used for extracting the antioxidant compounds from the legumes. Moreover, the differences in antioxidant activities measured via methods like DPPH, ABTS, FRAP, and CUPRAC for the same legume can be attributed to various factors related to the specific characteristics of each assay [72]. Each method targets different mechanisms of antioxidant action; for instance, DPPH and ABTS assess radical scavenging ability, while FRAP and CUPRAC evaluate the reducing power of antioxidants [73]. The amount and type of phenolics and flavonoids influence the antioxidant content across different assays. Higher levels of phenolic compounds are often associated with enhanced capacity to neutralize free radicals and protect against oxidative stress.

Processing methods further impact the bioavailability and stability of antioxidants. Cooking methods, such as boiling or soaking, may lead to the leaching of water-soluble phenolics, reducing the TPC and consequently the antioxidant activity [74]. Fermentation often increases levels of bioactive compounds, enhancing the antioxidant activity due to microbial metabolism [38]. Germination is known to boost both total phenolic and flavonoid content, further increasing the antioxidant capacity [36,74].

Consequently, the inherent differences in the chemical nature of the antioxidants, present in the legume, and the specific reactions evaluated by each assay result in variations between the measured antioxidant activities.

In this study, most of the analyzed legumes demonstrated high antioxidant capacity, as assessed via the ABTS and CUPRAC methods, likely attributed to the presence of phenolics and the number and positioning of hydroxyl groups on the aromatic ring, as well as the types of substituents in additional structures that can function as ligands.

**Figure 2:**
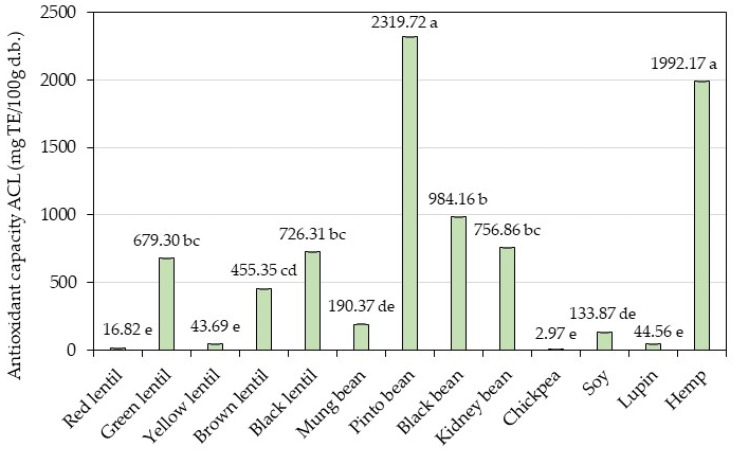
Antioxidant capacity using the Photochem device in an ACL (lipid-soluble antioxidant capacity) system. Bars with different letters represent a significant difference between samples (*p* < 0.05).

The inhibition of the photochemiluminescence (PCL) of luminol (5-amino-2,3-dihydrol, 4-phthalazinedione) in methanol by constituents of the phenolic-containing sample extracts was monitored using the Photochem system. The results are presented in Figure 2. The lipid-soluble antioxidant capacity data of the 13 analyzed samples were found to be between 2.97 ± 0.29 and 2319.72 ± 1.95 mg TE/100 g d.b.

The highest value was determined in pinto bean, followed by hemp and black bean. Chickpea showed the lowest value of antioxidant capacity in lipidic system. With respect to lentil samples, the highest value of ACL was presented by black lentil, followed by green lentil. Red lentil exhibited the lowest antioxidant capacity. In aqueous–organic extracts of bean samples, ACL values were between 190.37 and 2319.72 mg/100 g d.b. Pinto bean showed the highest value of antioxidant capacity, followed by black bean and kidney bean.

A high correlation coefficient was observed between the TPC and DPPH (r = 0.8133), TPC and FRAP (r = 0.8528), TPC and CUPRAC (r = 0.9425), and TPC and ACL (r = 0.8261) methods.

Strong positive correlations were observed between the DPPH and FRAP, DPPH and CUPRAC, DPPH and ACL, FRAP and CUPRAC, FRAP and ACL, and CUPRAC and ACL methods, respectively, with correlation coefficients exceeding 0.7 (Table 6).

### 3.6. Calculation of the Relative Antioxidant Capacity Index

**Figure 3:**
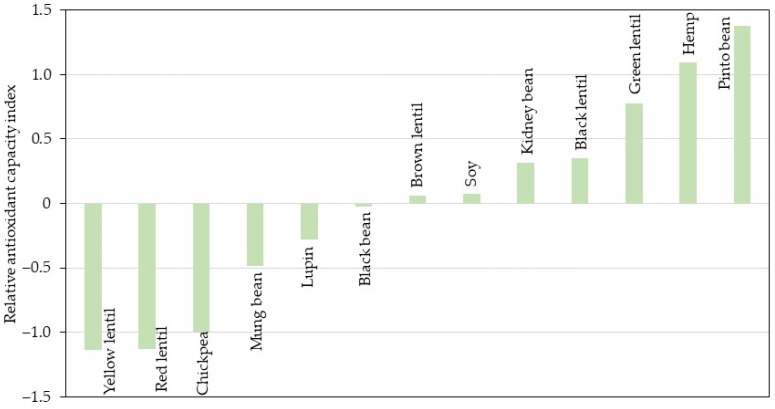
Relative antioxidant capacity index in the legumes and hemp samples.

Based on the procedure described by Sun and Tanumihardjo [31], the relative antioxidant capacity index (RACI) was calculated as a statistical perspective by integrating food antioxidant capacity data determined via the several methods used. The RACI of each sample was calculated as the mean of the standard scores transformed from the raw data generated with different chemical methods. The difference in units and variances in the raw data had no influence on the RACI. The RACI is a scientific combination of data from different chemical methods with no unit limitation and no variance among methods and allows for a comparison of the food antioxidant capacity [31]. The ranking of the antioxidant capacities of the analyzed samples based on RACI is presented in Figure 3.

**Figure 4:**
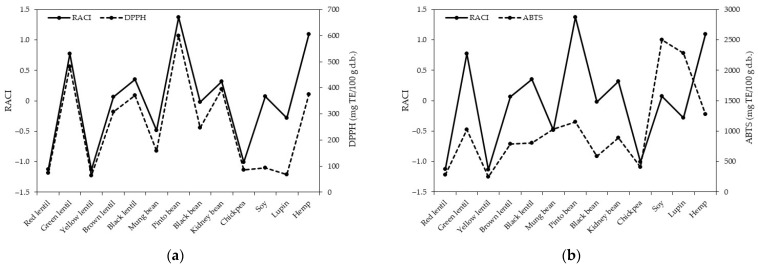

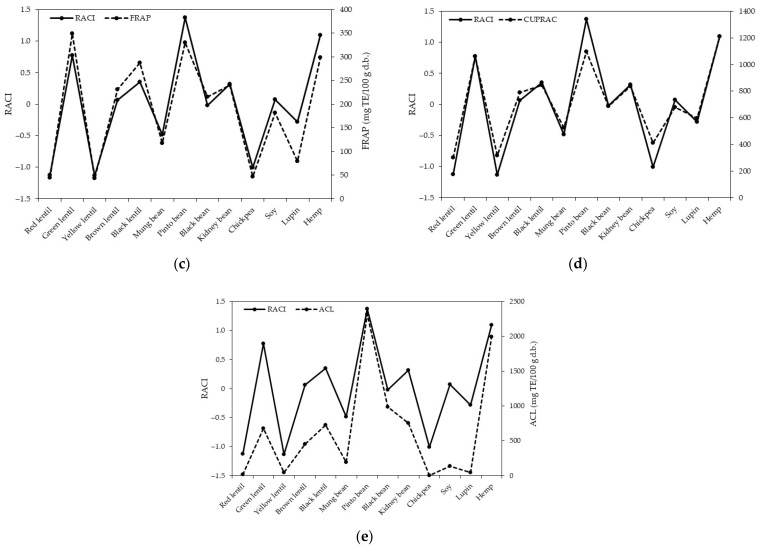
Correlation between RACI and antioxidant capacity assays: (**a**) RACI and DPPH; (**b**) RACI and ABTS; (**c**) RACI and FRAP; (**d**) RACI and CUPRAC; (**e**) RACI and ACL.

Based on the RACI, pinto bean, hemp, and green lentil had the highest antioxidant capacity, while yellow lentil, red lentil, and chickpea presented the lowest antioxidant capacity. Furthermore, the RACI value was correlated with the antioxidant capacity assays (Figure 4). The RACI value was highly correlated with DPPH (r = 0.8888; *p* < 0.05), FRAP (r = 0.9435; *p* < 0.05), CUPRAC (r = 0.9811; *p* < 0.05), and ACL (r = 0.8564; *p* < 0.05). However, no good correlation was found between RACI and ABTS (r = 0.3613; *p* > 0.05). Therefore, this approach was proven to be an appropriate way to quantify food antioxidant capacity as it can represent each chemical method.

### 3.7. Determination of Color Parameters

**Figure 5:**
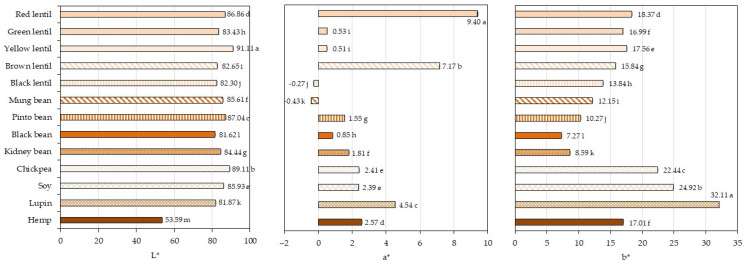
Powdered legume colors in the CIE L*a*b* color coordinates are shown as bar length and bar color. The values are presented as mean values ± standard deviation (n = 10). The values followed by different letters indicate significant differences between samples (*p* < 0.05).

The color is an important parameter that will reflect the quality of the developed legume-based food products, being the first attribute that consumers will assess. The color measurement results in the CIE L*a*b* coordinates, obtained from various legumes, are presented in Figure 5, while an image of the powders is shown in Figure S1. The color parameters varied between the legume samples, and significant differences (*p* < 0.05) were found. Lightness value L* (where L* = 0 is black; L* = 100 is white) was the highest for yellow lentil. The darkest sample was hemp. The darkening is attributed to a higher mineral content. Lightness was negatively correlated with the ash content (r = −0.8427; *p* < 0.05), meaning that darker legumes (lower L*) had more minerals. The larger the a* value, the redder the color; and the smaller the a* value, the greener the color. A higher a* value denoted more redness in the legumes samples, especially for red lentil, brown lentil, and lupin. The negative a* values for black lentil and mung bean indicated greenness in these legumes. The larger the b* value, the yellower the color; and the smaller the b* value, the bluer the color. The b* values indicated the presence of yellow pigments in all the legume samples with a wider range of yellowness: lupin (32.11) and black bean (7.27). The greatest yellowness was noted for lupin, soy, chickpea, red lentil, and yellow lentil. Greater a* and b* values indicate that the yellowness and redness are more intense. Chroma (C*), representing color saturation or intensity, had lower values for all the bean samples (7.3 for black, 8.8 for kidney, 10.4 for pinto, and 12.2 for mung beans), followed by lentil samples (13.8 for black, 17.0 for green, 17.4 for brown, 17.6 for yellow, and 20.6 for red lentils) and hemp (17.2). The highest chroma was for chickpea (22.6), soy (25.0), and lupin (32.4).

Correlation analyses between color parameters and TPC and TFC and antioxidant capacity only revealed significant negative correlations between L* and TPC (r = −0.59; *p* < 0.05), L* and CUPRAC (r = −0.6178; *p* < 0.05), L* and ACL (r = −0.5575; *p* < 0.05), b* and DPPH (r = −0.5898; *p* < 0.05), C* and DPPH (r = −0.6031; *p* < 0.05). These results indicate that dark legume samples contain more TPC and less antioxidant capacity (CUPRAC and PCL assays), while yellow legumes have less antioxidant capacity (DPPH assay). There were no correlations between TFC and the color values of legume powders. Similar findings have been reported in a previous study on food legumes [75].

## 4. Conclusions

This study provides an evaluation and comparison of different legumes and hemp samples from a nutritional point of view. The protein content was the highest in hemp, soy, and lupin (42.2–47.2% d.b.), and lowest in mung bean (22.4% d.b.). The content of glutamic and aspartic acid was the highest among the amino acids, while methionine and cysteine were the lowest. Based on the PDCAAS values, soy, chickpea, and mung bean are generally of high quality. Of the legumes included in the analysis, mung bean, black bean, kidney bean, chickpea, and soy met the amino acids requirements as recommended by FAO. The other legumes were below the FAO requirements for sulfur amino acids, while hemp did not reach the lysine requirement.

Color analysis showed that L* was negatively correlated with the ash content, TPC, CUPRAC, and ACL; while b* and C* were negatively correlated with DPPH.

The remarkable aspects of these legumes include their high phenolic compound content, which significantly contributes to their antioxidant capacity and overall health benefits. The strong correlation of photochemiluminescence analysis with established antioxidant assays like DPPH, FRAP, and CUPRAC indicates that these legumes are effective in neutralizing free radicals, thus helping to combat oxidative stress. Additionally, the presence of bioactive components in legumes can support various health functions, such as reducing the risk of chronic diseases, improving heart health and enhancing immune function. Their nutritional profile is also noteworthy as legumes are typically rich in proteins, fiber, vitamins, and minerals, making them valuable functional foods that can be easily incorporated into a balanced diet. Overall, the combination of their antioxidant properties and nutrient density makes legumes a remarkable food source with great potential in promoting health and well-being.

Because of the variation in the amino acid content and in the composition between various legume sources, we emphasize the need for legume combinations or blends of legumes with cereals to counteract the deficiency in sulfur amino acids.

The legume screening performed in this study from a nutritional point of view represents a first step towards selecting optimal legume combinations for inclusion in the development of new food products (e.g., bakery and vegan alternative products).

## Figures and Tables

**Table 1:** Chemical composition of the analyzed legumes and hemp samples (reported as g/100 g dry basis).

Samples	Protein	Fat	Ash	Starch	Fiber
Red lentil	28.22 ± 0.49 f	1.16 ± 0.06 g	2.64 ± 0.05 h	53.57 ± 0.15 a	10.01 ± 0.05 k
Green lentil	28.95 ± 0.18 e	0.77 ± 0.10 ij	3.11 ± 0.03 f	28.76 ± 0.04 h	33.44 ± 0.20 a
Yellow lentil	28.19 ± 0.36 f	1.27 ± 0.01 fg	2.45 ± 0.02 i	37.66 ± 0.11 f	25.19 ± 0.05 d
Brown lentil	28.37 ± 0.12 ef	0.66 ± 0.02 j	2.51 ± 0.05 i	50.20 ± 0.30 b	12.71 ± 0.31 j
Black lentil	33.23 ± 0.59 d	0.80 ± 0.02 hi	2.81 ± 0.05 g	39.79 ± 0.04 e	18.64 ± 0.16 g
Mung bean	22.36 ± 0.25 i	0.92 ± 0.02 h	3.94 ± 0.05 d	50.24 ± 0.22 b	17.77 ± 0.12 h
Pinto bean	23.42 ± 0.02 h	1.41 ± 0.01 e	4.21 ± 0.01 c	39.52 ± 0.17 e	26.33 ± 0.09 c
Black bean	24.79 ± 0.30 g	1.21 ± 0.01 fg	4.18 ± 0.01 c	47.65 ± 0.24 c	16.68 ± 0.08 i
Kidney bean	28.34 ± 0.14 f	1.32 ± 0.01 ef	3.64 ± 0.03 e	36.38 ± 0.12 g	24.76 ± 0.11 d
Chickpea	24.80 ± 0.12 g	5.70 ± 0.01 d	2.52 ± 0.01 i	41.90 ± 0.14 d	19.44 ± 0.17 f
Soy	43.86 ± 0.30 b	24.36 ± 0.10 a	5.25 ± 0.02 b	15.19 ± 0.08 j	9.70 ± 0.01 k
Lupin	42.24 ± 0.02 c	7.45 ± 0.13 c	3.10 ± 0.02 f	16.57 ± 0.11 i	28.59 ± 0.11 b
Hemp	47.25 ± 0.17 a	10.42 ± 0.02 b	8.50 ± 0.12 a	7.28 ± 0.04 k	23.46 ± 0.23 e

Values are expressed as mean ± standard deviation (n = 3). Different letters in each column show significant difference between samples (*p* < 0.05).

**Table 2:** Amino acids composition (g/100 g protein) of the legumes and hemp samples.

**Samples**	**His**	**Ile**	**Leu**	**Lys**	**Met**	**Cys**	**Thr**	**Val**	
Red lentil	2.93 ± 0.08 a	4.81 ± 0.16 a	8.43 ± 0.08 a	7.68 ± 0.10 a	0.87 ± 0.06 f	0.78 ± 0.04 de	4.54 ± 0.06 a	5.06 ± 0.08 a	
Green lentil	2.37 ± 0.04 f	3.92 ± 0.12 d	6.74 ± 0.31 g	6.33 ± 0.08 de	0.83 ± 0.02 f	0.75 ± 0.02 def	3.55 ± 0.06 g	3.97 ± 0.10 i	
Yellow lentil	2.58 ± 0.02 cd	4.42 ± 0.07 bc	7.52 ± 0.21 cde	6.87 ± 0.12 b	0.94 ± 0.06 ef	0.75 ± 0.06 def	3.87 ± 0.10 f	4.56 ± 0.06 cde	
Brown lentil	2.43 ± 0.02 ef	4.01 ± 0.06 d	6.85 ± 0.02 fg	6.14 ± 0.04 ef	0.82 ± 0.04 f	0.75 ± 0.02 def	3.44 ± 0.02 g	4.10 ± 0.04 hi	
Black lentil	2.60 ± 0.08 cd	4.48 ± 0.10 ab	7.90 ± 0.09 bc	6.74 ± 0.20 bc	0.84 ± 0.05 f	0.65 ± 0.08 ef	4.08 ± 0.05 de	4.74 ± 0.11 bc	
Mung bean	2.53 ± 0.05 de	4.13 ± 0.10 cd	7.43 ± 0.10 de	6.56 ± 0.12 cd	1.32 ± 0.03 c	1.02 ± 0.03 b	3.39 ± 0.08 g	4.63 ± 0.08 bcd	
Pinto bean	2.61 ± 0.03 bcd	4.49 ± 0.07 ab	7.85 ± 0.14 bcd	6.61 ± 0.07 bc	1.07 ± 0.03 e	0.88 ± 0.03 cd	4.45 ± 0.07 ab	4.77 ± 0.03 b	
Black bean	2.40 ± 0.03 ef	4.15 ± 0.21 cd	7.23 ± 0.09 ef	5.97 ± 0.03 f	1.40 ± 0.07 c	0.97 ± 0.03 bc	4.12 ± 0.04 cd	4.32 ± 0.03 fg	
Kidney bean	2.52 ± 0.02 de	4.00 ± 0.10 d	7.31 ± 0.10 e	6.14 ± 0.10 ef	1.31 ± 0.06 c	1.07 ± 0.08 b	3.98 ± 0.07 def	4.20 ± 0.10 gh	
Chickpea	2.48 ± 0.03 def	4.13 ± 0.17 cd	7.21 ± 0.13 ef	5.42 ± 0.07 g	1.60 ± 0.09 b	1.49 ± 0.03 a	3.85 ± 0.07 f	4.40 ± 0.03 ef	
Soy	2.70 ± 0.04 bc	4.80 ± 0.04 a	8.39 ± 0.10 a	6.18 ± 0.04 ef	1.11 ± 0.04 de	1.39 ± 0.02 a	4.33 ± 0.05 b	4.50 ± 0.07 def	
Lupin	3.02 ± 0.03 a	4.59 ± 0.05 ab	8.10 ± 0.04 ab	4.88 ± 0.09 h	1.26 ± 0.08 cd	0.63 ± 0.01 f	4.27 ± 0.04 bc	4.17 ± 0.04 ghi	
Hemp	2.74 ± 0.07 b	4.12 ± 0.06 cd	7.12 ± 0.17 efg	4.03 ± 0.01 i	2.05 ± 0.07 a	1.45 ± 0.07 a	3.93 ± 0.02 ef	4.66 ± 0.03 bcd	
FAO *	1.6	3.0	6.1	4.8	2.3		2.5	4.0	
								
**Samples**	**Phe**	**Tyr**	**Glu**	**Asp**	**Arg**	**Ala**	**Gly**	**Pro**	**Ser**
Red lentil	5.79 ± 0.06 a	2.98 ± 0.06 b	21.25 ± 0.06 c	13.91 ± 0.10 a	9.43 ± 0.18 d	4.77 ± 0.08 a	4.46 ± 0.10 bc	4.98 ± 0.39 bcd	5.89 ± 0.10 a
Green lentil	4.68 ± 0.04 de	2.42 ± 0.02 d	15.92 ± 0.02 j	10.43 ± 0.02 i	7.27 ± 0.15 g	3.82 ± 0.06 d	3.57 ± 0.02 ghi	3.25 ± 0.07 f	4.68 ± 0.04 g
Yellow lentil	5.19 ± 0.15 b	2.61 ± 0.02 c	18.11 ± 0.06 g	11.47 ± 0.04 g	8.94 ± 0.06 e	4.20 ± 0.06 bc	3.90 ± 0.10 ef	4.26 ± 0.17 de	5.18 ± 0.06 e
Brown lentil	4.66 ± 0.07 de	2.38 ± 0.02 de	16.88 ± 0.08 i	11.03 ± 0.14 h	8.34 ± 0.10 f	3.81 ± 0.07 d	3.61 ± 0.04 ghi	3.86 ± 0.11 ef	4.75 ± 0.06 fg
Black lentil	5.29 ± 0.09 b	2.73 ± 0.08 c	19.50 ± 0.09 e	12.97 ± 0.17 c	10.48 ± 0.15 b	4.32 ± 0.05 b	4.09 ± 0.05 de	4.41 ± 0.39 cde	5.47 ± 0.07 cd
Mung bean	5.72 ± 0.08 a	2.26 ± 0.03 e	18.49 ± 0.05 f	11.71 ± 0.03 f	6.24 ± 0.05 h	4.20 ± 0.03 bc	3.52 ± 0.05 hi	4.81 ± 0.36 bcd	4.87 ± 0.03 f
Pinto bean	5.65 ± 0.09 a	2.62 ± 0.03 c	17.56 ± 0.03 h	13.08 ± 0.05 c	5.92 ± 0.15 h	4.05 ± 0.05 c	3.66 ± 0.07 gh	4.84 ± 0.35 bcd	5.86 ± 0.03 a
Black bean	5.17 ± 0.03 bc	2.60 ± 0.04 c	15.25 ± 0.05 k	12.02 ± 0.03 e	6.03 ± 0.03 h	3.79 ± 0.03 d	3.52 ± 0.11 hi	4.55 ± 0.21 cde	5.39 ± 0.03 d
Kidney bean	5.33 ± 0.12 b	2.44 ± 0.06 d	16.77 ± 0.02 i	12.11 ± 0.02 e	6.24 ± 0.07 h	3.69 ± 0.02 d	3.44 ± 0.06 i	3.90 ± 0.24 ef	5.59 ± 0.06 bc
Chickpea	5.83 ± 0.11 a	2.36 ± 0.04 de	18.22 ± 0.03 g	12.38 ± 0.07 d	9.97 ± 0.14 c	4.29 ± 0.07 b	3.77 ± 0.07 fg	4.76 ± 0.45 bcd	5.22 ± 0.04 e
Soy	5.67 ± 0.07 a	3.59 ± 0.04 a	22.56 ± 0.02 b	13.45 ± 0.04 b	8.54 ± 0.05 f	4.67 ± 0.12 a	4.28 ± 0.04 cd	6.42 ± 0.14 a	5.74 ± 0.04 ab
Lupin	4.62 ± 0.03 e	3.60 ± 0.06 a	27.61 ± 0.06 a	12.41 ± 0.06 d	12.70 ± 0.11 a	4.12 ± 0.03 c	4.76 ± 0.03 a	5.53 ± 0.21 b	5.74 ± 0.01 ab
Hemp	4.91 ± 0.12 cd	3.07 ± 0.09 b	20.66 ± 0.03 d	11.97 ± 0.06 e	12.61 ± 0.10 a	4.75 ± 0.12 a	4.64 ± 0.09 ab	5.24 ± 0.2 3 bc	5.52 ± 0.06 cd
FAO *	4.1								

Values are expressed as mean ± standard deviation (n = 3). Different letters in each column show significant difference between samples (*p* < 0.05). * FAO recommended amino acid scoring pattern for older children, adolescents, adults [24].

**Table 3:** Amino acid scores (AAS), protein digestibility corrected amino acid score (PDCAAS), and essential amino acid index (EAAI) of the legumes and hemp samples.

Samples	AAS *								PDCAAS	EAAI **
	His	Ile	Leu	Lys	Cys + Met	Phe + Tyr	Thr	Val		
Red lentil	1.83	1.60	1.38	1.60	**0.72**	2.14	1.82	1.26	0.57	84.39
Green lentil	1.48	1.31	1.11	1.32	**0.68**	1.73	1.42	0.99	0.53	69.28
Yellow lentil	1.61	1.47	1.23	1.43	**0.74**	1.90	1.55	1.14	0.57	76.47
Brown lentil	1.52	1.34	1.12	1.28	**0.68**	1.72	1.38	1.03	0.53	69.48
Black lentil	1.63	1.49	1.30	1.40	**0.65**	1.96	1.63	1.18	0.50	76.86
Mung bean	1.58	1.38	1.22	1.37	**1.02**	1.95	1.35	1.16	0.77	77.26
Pinto bean	1.63	1.50	1.29	1.38	**0.85**	2.02	1.78	1.19	0.60	80.54
Black bean	1.50	1.38	1.18	1.24	**1.03**	1.89	1.65	1.08	0.70	76.73
Kidney bean	1.58	1.33	1.20	1.28	**1.03**	1.89	1.59	1.05	0.73	76.62
Chickpea	1.55	1.38	1.18	1.13	1.34	2.00	1.54	**1.10**	0.80	78.62
Soy	1.69	1.60	1.38	1.29	**1.09**	2.26	1.73	1.13	0.92	84.39
Lupin	1.89	1.53	1.33	1.02	**0.82**	2.00	1.71	1.04	0.64	77.40
Hemp	1.71	1.37	1.17	**0.84**	1.52	1.94	1.57	1.17	0.68	78.25

* AAS values were calculated based on the recommended amino acid scoring pattern for older children, adolescents, and adults (g/100 g protein): 1.6 (His),3.0 (Ile), 6.1 (Leu), 4.8 (Lys), 2.3 (sulfur amino acids: Met + Cys), 4.1 (aromatic amino acids: Phe + Tyr), 2.5 (Thr), and 4.0 (Val) [24]. Highlighted values (bold) represent the lowest AAS. ** EAAI values were calculated based on the standard egg protein (g/100 g protein): 2.2 (His), 5.4 (Ile), 8.6 (Leu), 7.0 (Lys), 5.7 (sulfur amino acids: Met + Cys), 9.3 (aromatic amino acids: Phe + Tyr), 4.7 (Thr), and 6.6 (Val) [26].

**Table 4:** Phenolic content and flavonoid content in analyzed legumes and hemp samples (reported as g/100 g dry basis).

Samples	TPC, mg GAE/100 g d.b	TFC, mg QE/100 g d.b.
Red lentil	85.89 ± 1.31 h	10.80 ± 0.21 g
Green lentil	327.31 ± 1.82 b	23.67 ± 0.22 d
Yellow lentil	69.00 ± 0.71 i	5.44 ± 0.01 h
Brown lentil	247.56 ± 1.97 e	19.67 ± 0.11 e
Black lentil	248.38 ± 2.19 e	28.57 ± 0.22 b
Mung bean	260.62 ± 2.36 d	45.47 ± 0.22 a
Pinto bean	425.19 ± 2.04 a	0.24 ± 0.11 j
Black bean	209.53 ± 2.04 g	5.93 ± 0.22 h
Kidney bean	302.05 ± 2.00 c	17.93 ± 0.20 f
Chickpea	91.64 ± 2.15 h	0.16 ± 0.01 j
Soy	242.06 ± 0.98 e	2.43 ± 0.23 i
Lupin	230.29 ± 0.60 f	26.22 ± 0.36 c
Hemp	432.56 ± 4.58 a	18.13 ± 1.01 f

Values are expressed as mean ± standard deviation (n = 3). Different letters on each column show significant difference between samples (*p* < 0.05).

**Table 5:** Antioxidant capacity of the legumes and hemp samples through different assays (reported as g/100 g dry basis).

Samples	DPPH	ABTS	FRAP	CUPRAC
	mg TE/100 g d.b.			
Red lentil	73.76 ± 2.30 j	285.59 ± 0.46 k	44.53 ± 1.74 j	298.43 ± 3.70 j
Green lentil	480.46 ± 0.62 b	1024.27 ± 0.56 e	351.62 ± 5.73 a	1060.27 ± 2.99 c
Yellow lentil	63.48 ± 2.32 k	245.97 ± 0.91 j	43.64 ± 1.36 j	314.41 ± 7.45 j
Brown lentil	307.38 ± 1.83 e	789.89 ± 0.97 h	228.79 ± 4.44 f	787.23 ± 4.84 e
Black lentil	371.94 ± 0.36 d	807.10 ± 1.51 g	285.04 ± 4.98 d	842.87 ± 4.70 d
Mung bean	158.73 ± 2.12 g	1036.50 ± 0.56 e	117.49 ± 2.28 h	524.62 ± 2.98 h
Pinto bean	600.00 ± 0.72 a	1149.57 ± 0.95 d	332.20 ± 4.21 b	1093.65 ± 3.90 b
Black bean	246.13 ± 0.26 f	587.82 ± 0.25 i	215.99 ± 3.08 f	684.19 ± 4.17 f
Kidney bean	395.63 ± 1.15 c	887.82 ± 0.28 f	241.27 ± 3.80 e	834.64 ± 5.98 d
Chickpea	84.62 ± 2.45 i	408.09 ± 0.58 j	46.53 ± 1.07 j	408.69 ± 7.88 i
Soy	93.42 ± 1.56 h	2499.54 ± 0.78 a	181.83 ± 1.90 g	678.86 ± 4.87 f
Lupin	68.19 ± 0.6 8 j,k	2276.06 ± 1.48 b	79.70 ± 1.12 i	596.60 ± 3.28 g
Hemp	375.08 ± 1.43 d	1273.99 ± 1.33 c	300.53 ± 4.88 c	1208.31 ± 6.02 a

Values are expressed as mean ± standard deviation (n = 3). Different letters in each column show significant difference between samples (*p* < 0.05).

**Table 6:** Correlation coefficients between different assays in antioxidant capacity analyzing.

Method	DPPH	ABTS	FRAP	CUPRAC	ACL
DPPH	1	−0.045	0.9276	0.8783	0.8221
ABTS	-	1	0.1656	0.3005	0.0354
FRAP	-	-	1	0.9457	0.7643
CUPRAC	-	-	-	1	0.8305
ACL	-	-	-	-	1

## Data Availability

The original contributions presented in the study are included in the article/Supplementary Materials, further inquiries can be directed to the corresponding author.

## Supplementary Materials

The following supporting information can be downloaded at https://www.mdpi.com/article/10.3390/foods13223528/s1: Figure S1: Image of the legumes and hemp powders.
