# Supplementary figures and images for: Screening of the Nutritional Properties, Bioactive Components, and Antioxidant Properties in Legumes
Source: Foods. 2024 Nov 5;13(22):3528. doi: 10.3390/foods13223528 (PMC11593270; doi:10.3390/foods13223528)

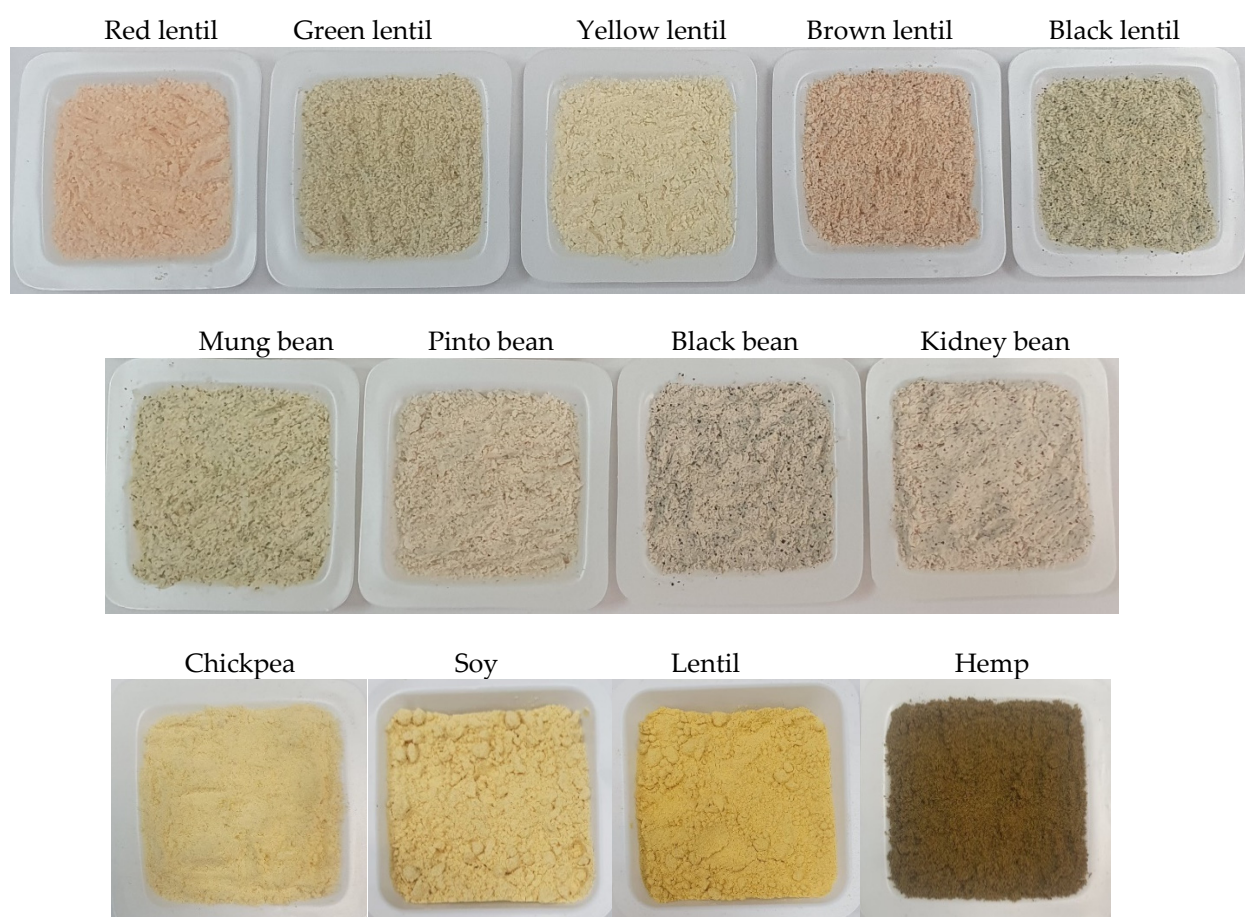

**Figure S1:** Image of the legumes and hemp powders.

Supplement: Supplementary file 1 [file foods-13-03528-s001.zip › foods-3283462-supplementary.pdf]
